# Extracellular vesicle release from intestinal organoids is modulated by *Apc* mutation and other colorectal cancer progression factors

**DOI:** 10.1007/s00018-019-03052-1

**Published:** 2019-04-26

**Authors:** Zsuzsanna Szvicsek, Ádám Oszvald, Lili Szabó, Gyöngyvér Orsolya Sándor, Andrea Kelemen, András Áron Soós, Krisztina Pálóczi, László Harsányi, Tamás Tölgyes, Kristóf Dede, Attila Bursics, Edit I. Buzás, Anikó Zeöld, Zoltán Wiener

**Affiliations:** 1 Department of Genetics, Cell and Immunobiology, Semmelweis University, Nagyvárad tér 4, 1089 Budapest, Hungary; 2 1st Department of Surgery, Semmelweis University, Üllői út 78, 1082 Budapest, Hungary; 3 Uzsoki Hospital, Uzsoki u. 29-41, 1145 Budapest, Hungary; 4 MTA-SE Immune-Proteogenomics Extracellular Vesicle Research Group, Nagyvárad tér 4, 1089 Budapest, Hungary

**Keywords:** Exosome, Adenoma, Cancer patient-derived organoid, Stroma, Tumor, Collagen

## Abstract

Extracellular vesicles (EVs) are membrane-surrounded structures that transmit biologically important molecules from the releasing to target cells, thus providing a novel intercellular communication mechanism. Since EVs carry their cargo in a protected form and their secretion is generally increased in tumorigenesis, EVs hold a great potential for early cancer diagnosis. By 3D culturing, we provide evidence that colorectal cancer (CRC) patient-derived organoids, representing a state-of-the-art established and essential approach for studying human CRC, is a suitable model for EV analysis. When testing the effects of major factors promoting CRC progression on EV release in the organoid model, we observed that *Apc* mutation, leading to uncontrolled Wnt activation and thus to tumorigenesis in the vast majority in CRC patients, critically induces EV release by activating the Wnt pathway. Furthermore, the extracellular matrix component collagen, known to accumulate in tumorigenesis, enhances EV secretion as well. Importantly, we show that fibroblast-derived EVs induce colony formation of CRC organoid cells under hypoxia. In contrast, there was no major effect of tumor cell-derived EVs on the activation of fibroblasts. Collectively, our results with CRC and *Apc*-mutant adenoma organoids identify *Apc* mutation and collagen deposition as critical factors for increasing EV release from tumors. Furthermore, we provide evidence that stromal fibroblast-derived EVs contribute to tumorigenesis under unfavorable conditions in CRC.

**Electronic supplementary material:**

The online version of this article (10.1007/s00018-019-03052-1) contains supplementary material, which is available to authorized users.

## Introduction

Colorectal cancer (CRC) is one of the most frequent causes of cancer-related death in the Western countries with a lifetime risk of approximately 5–6% [1]. In the large proportion of patients, *APC* inactivation is a central initializing mutation in CRC tumorigenesis. This results in the continuous activation of the Wnt pathway, which leads to increased cell proliferation and loss of cell differentiation by intestinal epithelial cells. Some of these adenomas progress then to invasive lesions (carcinomas) by the accumulation of further mutations [2, 3]. In addition, changes in the extracellular matrix composition, such as the accumulation of collagen fibers [4], and signals from stromal cells function as major drivers in CRC tumor progression and metastasis formation [5].

Extracellular vesicles (EVs) are membrane-surrounded structures that represent a novel way of intercellular communication by delivering biologically important molecules, such as miRNAs, proteins, and lipids from the releasing to the target cells. EVs are heterogeneous considering their biogenesis, size, molecular cargo, specific markers, and functions [6–9]. Exosomes are EVs (30–100 nm) of endosomal origin, derived from the multi-vesicular bodies (MVB) and released from cells upon fusion of the MVBs with the plasma membrane. Microvesicles (MVs) are shed directly from the plasma membrane and the larger apoptotic bodies (1–5 µm) are released by apoptotic cells [10]. Since EVs are present in body fluids, they may hold a great promise in early cancer diagnosis. This assumption is based on the fact that tumor cells release EVs at a higher level compared to normal cells [11] and that cancer cell-derived EVs carry tumor-specific molecules as cargo in a membrane-surrounded, protected milieu. However, EV production and their molecular composition are highly dependent on the culture conditions, isolation methods, and external factors critically influencing both parameters [12]. Most of the published works focusing on EVs have so far used traditional 2D cell cultures in CRC. Unfortunately, the classical 2D tumor cell lines that have been cultured for a long time are derived from a limited cell population of cancer patients, and are highly selected upon setting up the 2D cultures. Thus, EV studies need a model system that better represents the in vivo situation in tumors. Furthermore, successful EV-based diagnostics critically depends on the amount of tumor-derived EVs in the body fluids. However, factors influencing EV production in CRC tumor cells are poorly characterized as yet.

The recently developed 3D organoid technology maintains the cellular and genetic heterogeneity of in vivo tissues and has proved to be so far the best ex vivo model of human cancers [13, 14]. By now, organoids have been successfully cultured from many mouse and human healthy and cancer tissues, including pancreas [15], small intestine [16], colon [17], liver [18], etc. under well-defined specific culture conditions. In our study, we provide evidence that the 3D organoid technology is suitable to study the production and functions of EVs in CRC. We prove that enrichment of extracellular matrix (ECM) in collagen type I and the Wnt pathway activating *Apc* mutation critically modify EV release by intestinal tumor organoids. Importantly, while we found no evidence of stromal fibroblast activation by cancer cell-derived EVs, fibroblast EVs increased the number of 3D organoids in hypoxia, highlighting their prominent role in CRC progression.

## Materials and methods

### Cell culture

HCT116, SW620, and HT29 CRC cell lines were a kind gift from Prof. Kari Alitalo, University of Helsinki, Finland. SW1222 cells and normal human colon fibroblasts (ATCC-1459) were from ECACC and ATCC, respectively. Thp-1 cells were from ATCC. Cells were cultured in DMEM high glucose (Gibco) supplemented with 10% FCS (Gibco), cyprofloxacine, antibiotic/antimycotic mix, and glutamine (Sigma). Cells were tested for mycoplasma contamination with Hoechst staining and only negative cultures were used in our experiments. Two days before EV collection, cells were washed with PBS three times and they were further cultured in either medium without FBS or containing 2.5% EV-free FBS. EV-free FBS was prepared by overnight ultracentrifugation at 100,000*g* or purchased from Gibco (exosome depleted One-Shot FBS). For 3D cultures, cells were treated with TrypLE (Gibco), embedded into Matrigel with 5000–10,000 cells depending on the cell line and cultured for 12-14 days.

### Producing and culturing Apc-mutant mouse organoids

The Pest County Government Office (Hungary) as the veterinary authority approved the maintenance and experiments with mice. Normal intestinal crypts from C57Bl/6J (Jackson Laboratory, 000664) or UBI-GFP mice (Jackson Laboratory, 004353) were isolated according to previously published methods [16]. Approximately 500 crypts were embedded into growth factor-reduced, phenol red-free Matrigel (Corning, 20 µl/well in 48-well plates) and cultured in small intestinal medium (SIM): advanced DMEM/F12 medium (Gibco) containing B27 and N2 supplements (Gibco), 10 mM HEPES (Sigma), 1 µM N-acetyl cysteine (Sigma), glutamine, penicillin/streptomycin, 100 ng/ml noggin (Peprotech), 50 ng/ml EGF (Peprotech), and 500 ng/ml mouse R-Spondin1 (R&D Systems). Crypts were mechanically split by vigorous pipetting and embedded into new Matrigel in every 4-5 days. In some experiments, organoids were added the GSK-3 inhibitor CHIR99021 dissolved in DMSO (3 µM, Tocris), 1% DMSO as control or 100 ng/ml murine Wnt3a (Peprotech) for 3 days before processing samples for immunostaining. In these studies, the EVs in the supernatants were detected by anti-CD81-coated beads after 3 days (see below).

sgRNA sequence for mouse *Apc* was published previously (sgRNA4, [19]) and cloned into lentiCRISPR v2 (Addgene 52961) into the BsmBI restriction sites according to Addgene’s instructions. *Apc*-mutant organoids were produced according to the previously published protocol [19] with some modifications. Briefly, mouse organoids were cultured in SIM with the GSK-3 inhibitor CHIR99021 (3 µM, Tocris) for 2 days, and organoids were then treated with TrypLE to obtain single cells. Cells were resuspended in SIM + CHIR99021 + 10 µg/ml Y-27632 (Rho-kinase inhibitor, Sigma) and plated in 48-well plate in 400 µl. They were transfected with JetPEI (Polyplus Transfection) using 1 µg plasmid and 2 µl JetPEI Reagent according to the manufacturer’s recommendations. Cells were centrifuged at 600 g at RT for 1 h and then further incubated for 4 h at 37 °C. After washing, cells were plated in Matrigel with SIM containing Y-27632. The Rho-kinase inhibitor, R-Spondin1, EGF, and noggin were removed 3 days after transfection and organoids with *Apc* mutation were selected for > 6 days without growth factors.

### Human organoid cultures

The Medical Research Council of Hungary approved all experiments involving human samples and informed consent was obtained from the patients. Tissue biopsies from patients were processed according to previously published methods [17, 20]. The isolated tissue pieces were embedded into Matrigel and cultured in human organoid medium (HOM) containing advanced DMEM/F12 with N2 and B27 supplements, 10 mM HEPES, 1 mM N-Acetyl-Cysteine, glutamine, penicillin/streptomycin, 500 nM A83-01 (Sigma), 10 uM SB202190-Monohydrochloride (Sigma), and 50 ng/ml EGF. In addition, the Rho-kinase inhibitor Y27632 (Sigma) was added after splitting and it was removed from medium at day 2. Organoids were isolated from Matrigel in every 4–6 days by centrifugation at 300 g for 5 min, mechanically disrupted, treated with TrypLE for 3–5 min and then after washing embedded into Matrigel again.

In some experiments, cell line-derived colonies or organoids were centrifuged at 300 g for 5 min, the Matrigel was removed and after washing steps with PBS, colonies or organoids were further cultured in 24-well suspension plates (Eppendorf). Hypoxia was generated with AnaeroGen 2.5L bags (Thermo Scientific). Hypoxic condition was checked by anaerobic indicator (Thermo Scientific, BR0055B). When testing the effect of EVs on fibroblasts, CRC organoids were cultured for 2 days and EVs from about 1 × 10^6^ cells were added to 1 × 10^5^ fibroblasts in 500 µl EV-free medium. In case of microarray experiments, EVs from 1.5 × 10^6^ cells were harvested and 2 × 10^6^ fibroblasts were used.

### Collagen-based organoid cultures

Organoids were centrifuged, washed with PBS twice, and embedded into collagen type I (from rat tail, Ibidi). To create 100 µl collagen matrix, 60 µl water, 10 µl 10 × MEM (Gibco), and 30 µl collagen I were mixed and the pH was set to 7.2 with 1 M NaOH. Collagen was then added to the organoids and they were cultured in droplets in 48-well plates in HOM. When removing cells, collagenase II (Sigma) was added to the medium for 30 min at 37 °C and cells were then centrifuged at 300 g for 5 min.

### qNano measurements

Culture supernatants were harvested after 48 h, centrifuged at 300*g* for 5 min, and the supernatant was then pelleted again at 2000 g for 20 min to remove cells and cell debris. Supernatant was applied to qNano (Izon) analysis. In case of SW1222 colonies, supernatants were directly measured after centrifugation at 300 g for 5 min on membranes with pore size of 100, 400, 800, or 2000 nm. Minimum 500 data points were collected or, when not possible, samples were measured for 5 min. Calibration beads CPC100B or CPC400G (Izon) were suspended in the same culture media.

### Human EV detection by anti-CD63 or anti-CD81-coated beads

Culture supernatants were collected, and after centrifuging at 300 g for 5 min and 2000 g for 20 min, EVs were bound to antibody-coated beads that had been blocked with 0.1% BSA (Sigma) in PBS for 30 min. According to our previous experiments, 20 µl or 6 µl of anti-CD63-coated beads (Thermo Fisher, 10606D) or anti-CD81-coated beads (Thermo Fisher, 10616D) were added to 200 µl supernatant, respectively. After overnight rotation at 4 °C, beads were magnetically separated, washed with Annexin binding buffer (BD Biosciences) three times and beads were labelled for 20 min. 10,000 beads were then measured on a FACSCalibur (BD) instrument. FITC-anti-CD81 (A15753, Molecular Probes), PE-anti-CD63 (SAB47000218, Sigma), and FITC Annexin V (SAB4700218, Sony) or APC Annexin V (Immunotools, 31490016) were used for detection (2 µl/sample). In all experiments, cells were counted with Burker chamber and results were normalized to cell number.

### Mouse EV detection by anti-CD81-coated beads

Anti-CD81 antibody (MA180209, Thermo Scientific) was bound to magnetic beads by Dynabeads Antibody Coupling Kit (Invitrogen) according to the manufacturer’s instructions, coupling 10 µl antibody to 2 mg beads. 2 µl of the antibody-coated beads was then applied to 200 µl supernatant similar to the human EV detection. EVs bound to beads were detected by PE-anti-CD81 antibody (MA517941, Thermo Scientific) and the results were normalized to cell number.

### Detecting PTK7 by flow cytometry

CRC organoids were removed from Matrigel and treated with TryPLE (Gibco) for 5–10 min to obtain single cells. Cells were then labelled with APC anti-PTK7 (Miltenyi Biotech) for 20 min and then centrifuged at 300 g for 5 min. 10,000 cells were measured with a flow cytometer (FACS Calibur, BD).

### EV detection by flow cytometry

EV detection by flow cytometry was carried out according to [21] with minor modifications. Briefly, cells and cell fragments were removed from cell culture supernatant by serial centrifugation at 300 g for 5 min and 2000 g for 20 min. 50 µl of supernatant was labelled with FITC Annexin V (Sony) for 30 min, and then, 200 µl Annexin Binding Buffer (BD Biosciences) was added. Large EVs were measured for 90 s, and then, samples were analyzed again after adding 0.1% Triton X-100 that disrupts EVs. Absolute counts were calculated using Count Check Microbeads (Sysmex Partec GmbH, Germany). Large EV absolute number with flow cytometer (FACS Calibur, BD) was determined with the following formula:

Number of vesicles in sample = (detected EV events in the EV gate—Triton X-100 resistant events)/number of events in bead gate) × Absolute count of Count Check beads in the tube × sample dilution.

### EV isolation

Cell or organoid culture supernatants were collected and serially centrifuged at 300 g for 5 min and 2000 g for 20 min. The supernatant was then further pelleted at 12,500 g for 20 min to obtain the large EV fraction. Small EVs were isolated with ultracentrifugation (UC) at 100,000*g* for 70 min at 4 °C. All EV fractions were washed with PBS and centrifuged or ultracentrifuged once more before usage. The large and small EVs were combined in functional tests. For miRNA detection, EVs were separated by UC or by the addition of anti-CD63 (120 µl/2 ml supernatant) and anti-CD81 coated beads (60 µl/2 ml supernatant) and EVs were lysed in Qiazol Lysis Reagent (Qiagen) after overnight incubation and washing steps with PBS (5 times). Alternatively, EV-derived total RNA was extracted with the ExoRNEasy Serum/Plasma Starter Kit (Qiagen) from 2 ml supernatant according to the manufacturer’s instruction.

### Proteomics analysis of EVs

CRC organoids were cultured in 20 µl Matrigel droplets in 200 µl HOM in 48-well plates and the supernatant was collected every second day. Samples collected from the same CRC organoid line were combined and EVs were separated from a total of 23 ml supernatant with differential UC. As a control, organoid-free Matrigel droplets were applied. EV pellet was resuspended in 80 µl water and proteins were extracted using repeated freeze–thaw cycles followed by miniaturized tryptic digestion as previously described [22]. Before the tryptic digestion, protein concentration was determined by the Micro BCA Protein Assay Kit (Thermo Scientific). Mass spectrometry of EV preparations was carried out according to [22]. Proteins present in the Matrigel controls were removed from organoid sample-derived lists of proteins and these lists were further analyzed.

### Testing the effects of fibroblast-derived EVs

Supernatant from fibroblasts (cultured in normoxia or hypoxia in HOM for 4 days) was collected, ultracentrifuged and the EV-containing pellet and EV-free supernatant were added to CRC organoid-derived cells. To produce CRC cells, CRC organoids were removed from Matrigel by centrifugation at 300 g for 5 min, treated with TrypLE until they were dissociated into single cells and they were washed with PBS before embedding into 20 µl Matrigel in 48-well plates (Eppendorf). EVs from about 500,000 fibroblasts were added to the Matrigel droplets. Colonies were counted 4–5 days after incubating in normoxia or hypoxia.

### Liposome production and characterization

High purity hydrogenated soy phosphocholine (HSPC, NC-21E, NOF Corporation, Japan) and cholesterol (C3045, Sigma) were used without further purification. Unilamellar liposome samples were prepared by the hydration and extrusion method. Briefly, 40 mg HSPC and 29 mg cholesterol were dissolved in 0.6 ml ethanol at 60 °C. 4 ml ultrapure water was added to the mixture and it was stirred for 10 min at 60 °C with a continuous nitrogen flow. The sample was then extruded ten times through polycarbonate filters (Nucleopore, Whatman Inc.) with 100 nm pore size using an LIPEX extruder (Northern Lipids Inc., Canada) at 60 °C. Finally, it was dialyzed against ultrapure water in a Slide-A-Lyzer™ MINI Dialysis Device (Thermo Fisher Scientific) for 24 h to remove residual traces of ethanol.

The size distribution of the liposome sample was characterized by dynamic light scattering (DLS). The DLS measurement was performed using a W130i apparatus (Avid Nano Ltd., High Wycombe, UK) and using a low volume disposable cuvette (UVette, Eppendorf Austria GmbH). The liposomes had a mean diameter of 105 nm.

### Transmission electron microscopy

After ultracentrifugation, the pellet was resuspended in 20 µl PBS and a 2 µl droplet was dried on a formfar-carbon coated 300 mesh grid (Electron Microscopy Sciences, USA) for 10 min. The EVs were then fixed with 4% glutaraldehyde for 10 min and the grid was washed with water three times. The EVs were stained with 2% phosphotungstic acid, they were allowed to dry at room temperature and imaged with a MORGAGNI 268D (FEI, The Netherlands) transmission electron microscope.

### Wound-healing assay

Human colon fibroblasts (ATCC-1459) were cultured in 24-well plate until confluence. A scratch was created with a p200 pipette tip in the cell monolayer. Cells were washed 3× with PBS to remove cell debris and 250 µl fresh medium (DMEM high glucose with 2.5% EV-free FBS, antibiotic/antimycotic mix, and glutamine) and 250 µl cell-culture supernatant after EV isolation (EV-free) was added to the cells. For the EV-treated cells, the EV pellet was resuspended in 20 µl PBS and added to the cells as well. Images were taken at 0, 12, 16, and 20 h with a Nikon Diaphot microscope. The cell-free areas were evaluated by the ImageJ software. In some experiments, EVs were isolated from CRC organoids cultured in hypoxia for 2 days.

### Whole-mount staining

Organoids were cultured in 4-well chamber slides (BD Biosciences), fixed in 4% PFA for 30 min, washed with PBS + 4% NaCl and blocked and permeabilized in blocking buffer (5% FBS, 0.2% BSA, 0.3% Triton X-100 in PBS) for 30 min. The following primary antibodies were applied at 4 °C overnight in blocking buffer: rat anti-vimentin (MAB2105, R&D Systems), rabbit anti-lumican (ab168348, Abcam), rabbit anti-active caspase-3 (AF835, R&D Systems), rabbit anti-Ki67 (Abcam, ab16667), rabbit anti-mucin2 (Santa-Cruz Biotechnology, sc-15334). After washing in PBS containing 0.3% Triton X-100 and 4% NaCl and overnight incubation with the secondary antibodies (Alexa Fluor 488 and 594, Thermo Fisher), the organoids were mounted in mounting medium containing DAPI (Thermo Fisher) and imaged with an Olympus FV500 confocal microscope.

### RNA isolation and RNA measurements

RNA and total RNA with small RNAs were isolated with the RNEasy Micro Kit and miRNeasy Micro Kit (Qiagen), respectively, according to the manufacturer’s instructions in 15 µl water. In some experiments, EV-derived miRNAs were obtained using the ExoRNEasy Serum/Plasma Starter Kit (Qiagen). RNA concentration was determined by NanoDrop.

For miRNA measurements, 2 µl total RNA (including small RNA) was reverse transcribed with the TaqMan^®^ Advanced miRNA cDNA Synthesis Kit (Thermo Fisher) according to the manufacturer’s description. PCR reactions were then carried out with TaqMan^®^ Fast Advanced Master Mix and TaqMan^®^ Advanced miRNA Assays (Thermo Fisher). A Ct value cutoff of 35 was considered as positive result and data are visualized as 35-Ct values. miRNAs and assay IDs are in Table S1.

For mRNA measurement, 0.5 µg total RNA isolated from organoids was reverse transcribed with the SensiFAST™ cDNA Synthesis Kit (Bioline). Quantitative PCR reactions using the SYBRGreen method were carried out with the SensiFAST™ SYBR^®^ Hi-ROX Kit (Bioline) on an ABI 7900HT Fast real-time PCR instrument in 384-well format in 5 µl volume. Results were calculated using the following protocol: relative expression level = 2^−ΔCt^, where ΔCt = Ct(gene of interest)—Ct(housekeeping gene). The primers used are listed in Table S1.

### Sequencing

cDNA was amplified with Phusion High Fidelity DNA Polymerase (Thermo Fisher) with the following primers: p53: TGAAGCTCCCAGAATGCCAG and CTTCAGGTGGCTGGAGTGAG (65 °C), KRAS: CCCAGGTGCGGGAGAGA and AGGCATCATCAACACCCTGT (65 °C). The DNA was then isolated from 2% agarose gel, purified by the Gel Purification Kit (Macherey–Nagel) and sequenced by the forward primers with Applied Biosystems 3500 Genetic Analyzer instrument (Life Technologies). Data were analyzed by the Chromas 2.6 software (Technelysium Pty Ltd).

### Microarray experiments

RNA quality was determined with the Bioanalyzer Pico Chip (Agilent) and analyzed on Agilent 4 × 44 K human whole genome expression microarrays. Data were processed by the Feature Extraction Software 12.0.3.1 (Agilent) and then imported into Chipster (www.chipster.csc.fi) and the standard Agilent one colour normalization method was applied. Preprocessing was carried out by filtering by standard deviation (percentage to filter out: 0.67, which is about 1 SD). Genes with expression difference were listed using empirical Bayesian paired *t* test with no *p* value adjustment methods. The pre-filtered genes were used for hierarchical clustering using the Pearson distance method. The microarray data have been submitted to the GEO database (www.ncbi.nlm.nih.gov/geo/) under the series accession number GSE114979.

### Gene set enrichment and survival analysis

The gene expression data set was transferred to the Gene Set Enrichment Analysis software (http://www.broadinstitute.org/gsea [23, 24] and the analysis was carried out with default parameters except that the “exclude smaller sets” was set to 20 and gene permutation was applied. We used a modified list of the kegg.v3.1.symbols gene set (http://www.broadinstitute.org/gsea/msigdb) with the addition of Wnt targets [25], intestinal stem cell-specific genes [26], and exosome biogenesis genes. FDR *q* value < 0.1 was regarded significant. The exosome biogenesis gene set was created based on published data and genes with proved function in MVB biogenesis and exosome secretion were selected. The gene set is shown in the Supplementary Information.

For survival analysis, the GSE17537 and GSE14333 data sets were used, both containing patient survival data as well. Expression levels were z-score transformed for all genes before carrying out Kaplan–Meier analysis and log rank test.

### Statistical analysis

Student’s paired or unpaired *t* test, Mann–Whitney *U* test, one-way ANOVA, and Tukey post hoc test or Kruskal–Wallis test with Dunn post hoc test were used with *p < 0.05, **p < 0.01, and ***p < 0.001 significance levels. For evaluation, Microsoft Excel, SPSS version 25, and Sigma Plot softwares were used.

## Results

### CRC organoid-derived EVs can be detected in cultures in 3D matrix

The SW1222 CRC cell line forms lumen-containing complex large colonies (megacolonies) in 3D matrix, containing both undifferentiated and differentiated cancer cells, similar to the in vivo tumors [27, 28]. To set up 3D cultures for EV detection, we used both basement membrane extract (BME) and Matrigel as 3D matrices. Tunable resistive pulse sensing (qNano) measurements, suitable for detecting particles with different sizes, showed that the background particle numbers of BME or Matrigel were negligible compared with 3D colonies (Fig S1a, b). Furthermore, we observed a higher concentration of smaller particles compared to larger ones in the supernatant of SW1222 colonies when using membranes of different pore sizes (Fig. S1c). Interestingly, we found a modestly increased colony-forming efficiency of SW1222 cells in Matrigel compared to BME (Fig. S1d), but there was no difference in particle production normalized to cell number (Fig. S1a). Importantly, SW1222 cells required EV-free fetal bovine serum (FBS) in the medium to form 3D colonies (Fig. S1d).

To identify and assess EVs from cell-culture supernatants, we used anti-CD63 or anti-CD81-coated magnetic beads and detected EVs by fluorescently labelled anti-CD63 or anti-CD81 antibodies and flow cytometry. CD63 and CD81 are generally regarded as EV markers and plasma membrane-derived larger EVs can often be labelled by Annexin V. As published previously [29], we observed a correlation between the cell number and the percentage of positive beads in two cell lines (Fig. S1e, f), furthermore, between the cell number and the particle number as well (Fig. S1g), showing that the bead-based method can be used for the semi-quantitative measure of EVs. Since the correlation between cell number and the positive beads was valid only above a threshold of cell number, we used similar cell numbers in all of our subsequent comparisons and we normalized all the results to cell number. Furthermore, we accepted experiments only when the sample with the lowest percentage of positive beads did not have the lowest cell number among the samples. Importantly, EVs released by SW1222 cells bound only to anti-CD63-coated beads, but not to beads covered by streptavidin, thus confirming the specificity of the system (Fig. S1h).

**Fig. 1:**
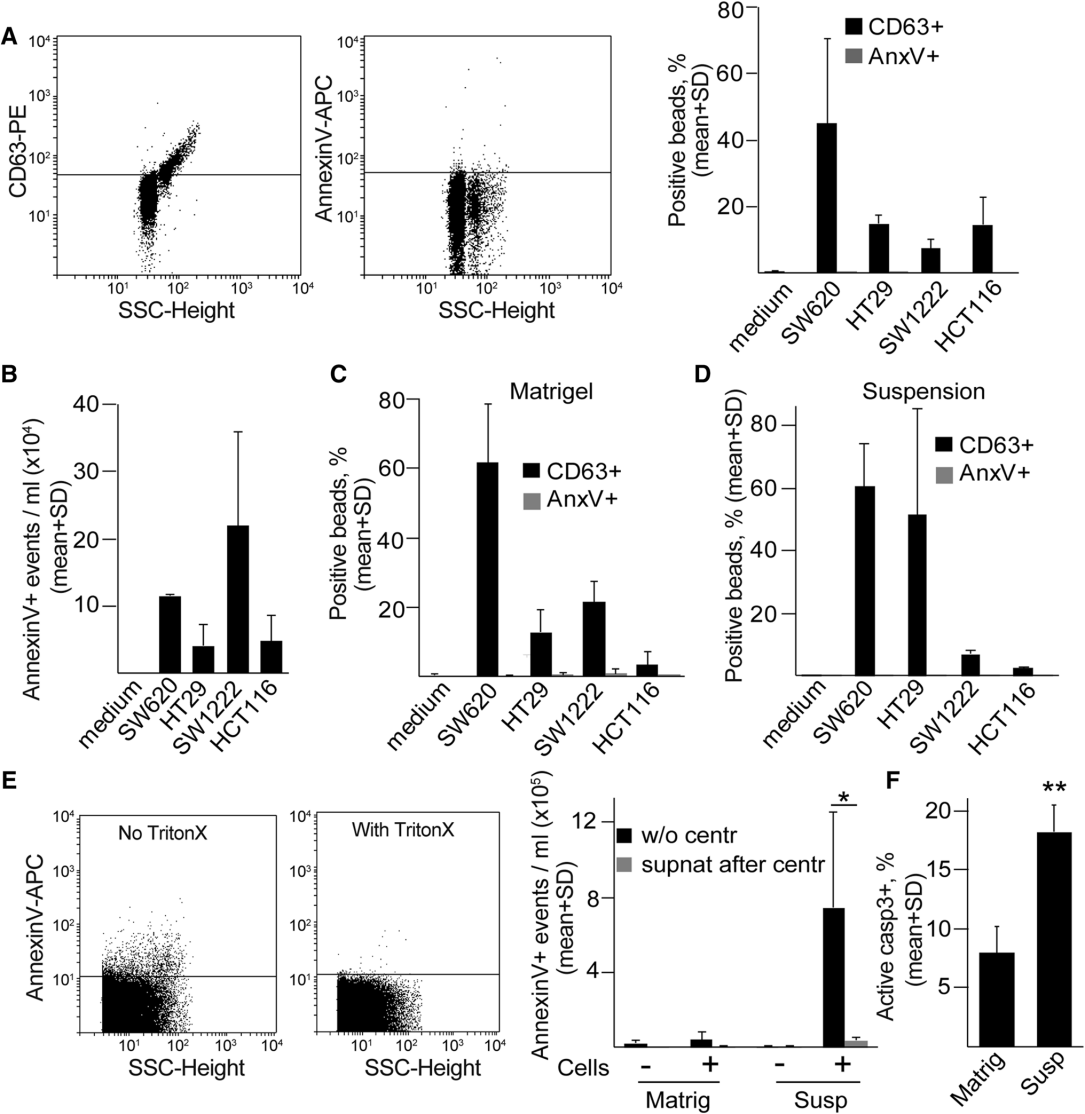
CD63 + EVs are released from 3D cultures. **a** Cells of the indicated cell lines were cultured 2D and EVs were captured by anti-CD63-coated beads, detected either by anti-CD63 or by Annexin V and the percentage of positive beads was measured with flow cytometry. In the left and middle panels, selected flow cytometric measurements from SW1222 cells are shown. Right panel: quantification of flow cytometry results. **b** Detecting Annexin V + events in the EV gate from 2D culture supernatants. Note that the largest EVs, containing apoptotic bodies, were removed by centrifugation before the measurements. In these experiments, EVs were directly measured by flow cytometry. (**c**–**d**) Percentage of positive beads from colonies cultured 3D in Matrigel (**c**) or after Matrigel culturing, the colonies were maintained in suspension (**d**) for 48 h. **e** Number of Annexin V + events in the supernatant of SW1222 cells, derived from 3D cultures or colonies maintained in suspension after removing from Matrigel (flow cytometry). Left and middle panels: selected flow cytometric measurements from suspension cultures. Annexin V + events were determined before and after the addition of Triton X-100 (see “Materials and methods”). Right panel: quantification of samples with or without cells and before or after centrifugation at 12,500 g for 20 min. Note that EVs are not detectable from the supernatant after centrifugation (*n* = 3, *t* test). **f** Percentage of active caspase-3 events in different culture conditions of SW1222 cells (flow cytometry, *n* = 3–5, *t* test). The percentage of positive beads was normalized to cell number

Next, we used other CRC cell lines that form 3D colonies with different morphologies (Fig. S2a). In 2D cultures, we detected both CD63 +/Annexin V–EVs by the bead-based method and Annexin V + large EVs by flow cytometry in all cell lines from the supernatant (Fig. 1a, b). Similarly, 3D colonies in Matrigel or colonies removed from 3D matrix and further cultured in suspension plates produced CD63 +/Annexin V–EVs (Fig. 1c, d). In a parallel experiment, we could detect CD81 + EVs as well (Fig. S2b, c). Interestingly, however, comparing the two culture conditions, larger Annexin V + EVs were present at a higher amount in suspension cultures compared to the 3D matrix-grown colonies (Fig. 1e). In these experiments, we detected Annexin V + events that were sensitive to Triton X-100 lysis using the previously published, flow cytometry-based method [30]. Collectively, these results suggest that colony-derived larger EVs can be detected at a much higher level in suspension. However, this culture condition results in the increase of active caspase-3 + apoptotic cells (Fig. 1f), thus showing that suspension condition is not optimal for the colonies.

**Fig. 2:**
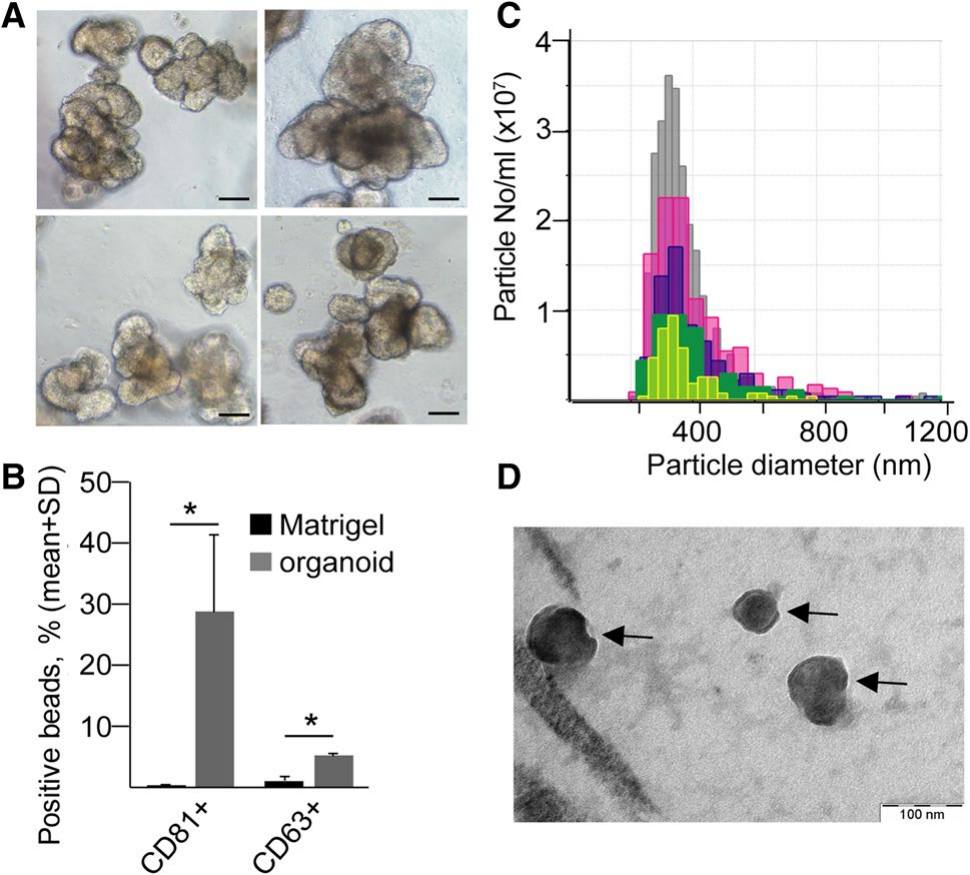
CRC patient organoid-derived EVs can be measured in 3D cultures. **a** Morphology of CRC patient-derived organoids (scale bars: 50 µm). **b** Percentage of EV-bound, anti-CD81 or anti-CD63-coated beads, detected by anti-CD81 or anti-CD63, respectively. Supernatants from 3D cultures without (Matrigel) or with organoids were processed after 48 h (flow cytometry, *n* = 3, *t* test). Positive bead percentage was normalized to 300,000 cells. **c** Size distribution and number of particles from five organoid cultures (qNano with 400 nm pore size). **d** Transmission electronmicroscopic image of CRC organoid-derived EVs (arrows), isolated by differential ultracentrifugation

The 3D organoid technology represents a superior model system, since these organoids are directly derived from CRC patients (Fig. 2a, Table S2), and similar to tumors, the cellular and genetic heterogeneity is a major hallmark of these organoids [31]. Importantly, all CRC patient-derived organoids produced CD63 + and CD81 + EVs in 3D cultures (Fig. 2b) and the presence of EVs in organoid culture supernatants was confirmed by qNano (Fig. 2c) and transmission electron microscopy as well (Fig. 2d).

We used three CRC patient-derived organoid lines (CRC1-3) in our further experiments. In CRC1-3, a subpopulation of the cells was highly positive for PTK7, a recently indentified marker for the stem-like cell population with high Wnt activity in CRC [32] (Fig. S2d). To investigate which pathways were mutant in these cultures, we tested their survival without R-Spondin1 or EGF or in the presence of nutlin-3 (see [33, 34]). Importantly, nutlin-3 inhibits p53 binding to the ubiquitin ligase MDM2; thus, cells with normal p53 undergo apoptosis. As expected, all cultures grew when the Wnt-agonist R-Spondin1 was absent, but none of them survived in the absence of EGF and they were insensitive to nutlin-3 (Table S3). These results agree with our sequencing data for KRAS and TP53 (Table S3). Thus, all three organoid cultures contained a continuously active Wnt and a mutant p53 pathway, but the KRAS pathway was not mutant.

### CRC organoid-derived EVs carry proteins and miRNAs

EVs are considered as transmission vehicles of proteins and miRNAs between cells. Our proteomic analysis of EVs from three CRC organoid lines showed that only about 45% of the detected proteins were present in all samples, highlighting the large variance between individual patient-derived organoid samples (Fig S3a and Tables S4, S5). To prove that CRC organoid-derived EVs carry miRNA in our model system, we measured 14 miRNAs in EVs isolated with three different methods. Whereas miR-484 or miR34a-5p were detected only by a commercial EV RNA isolation kit, most other miRNAs were present both in case of this kit or differential ultracentrifugation, although at different amounts (Fig. S3b, c). Interestingly, when using antibody-coated beads, we could detect only 8 miRNAs out of the 14 measured (Fig. S3d) and this isolation method resulted in the lowest unspecific background. Thus, similar to previously published results from 2D cultures, CRC organoid-derived EVs carry proteins and miRNAs as cargo; however, the EV isolation method largely influences the detected profile and there is a high variation among samples.

### The tumor promoting collagen I ECM protein induces EV release from CRC organoids

**Fig. 3:**
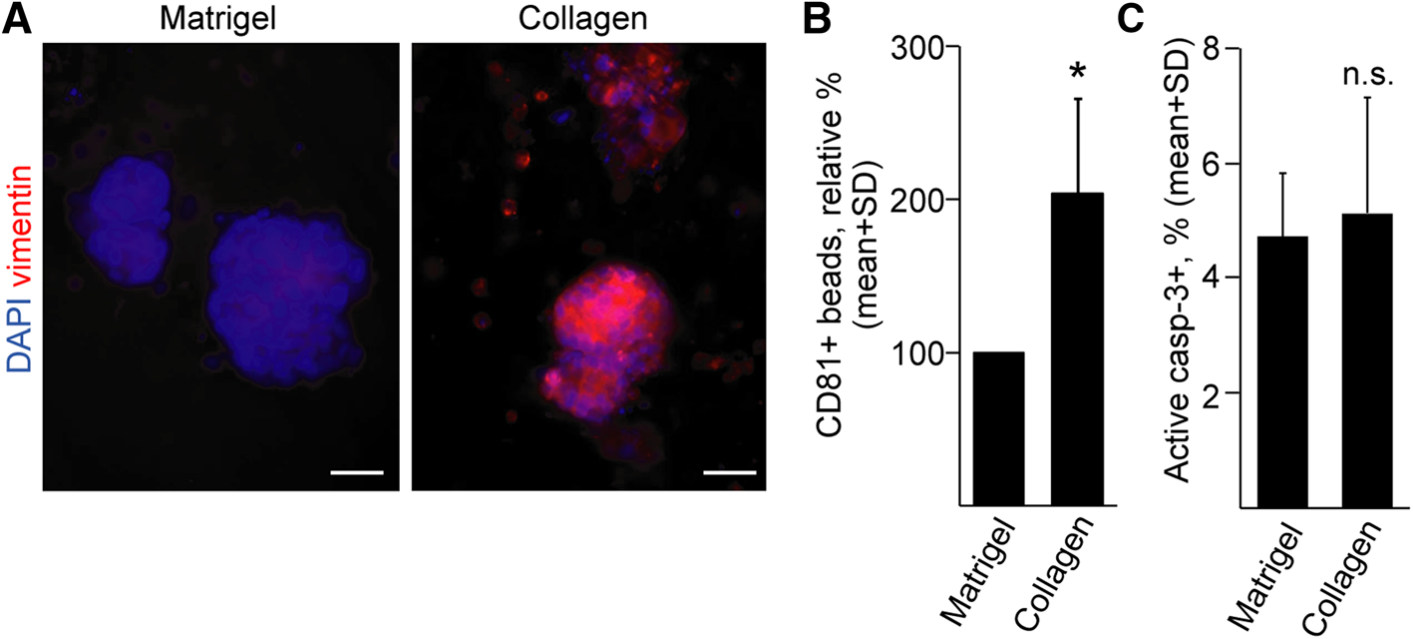
Replacing Matrigel to collagen I results in an elevated EV release from CRC organoids. **a** Vimentin whole-mount immunostaining of one of the CRC organoid lines, cultured in Matrigel or collagen for 48 h. **b** Relative percentage of CD81 + beads derived from human CRC organoids. Pairwise experiments were carried out and in each experiment collagen data normalized to cell number was compared to the normalized Matrigel-derived data (*n* = 4). The percentage of positive beads in Matrigel and collagen cultures without organoids was < 1%. **c** Quantification of active caspase-3 + apoptotic cells in CRC organoids. Images were taken from whole-mount stainings and the proportion of positive cells was counted. Ten organoids were counted from each CRC organoid line and their average was then used for evaluation. Scale bars: 20 µm. Paired (**b**) or unpaired (**c**) *t* tests were done

Having proved that organoids produce EVs in 3D matrices, we wanted to apply this system to find factors that influence EV release. Changes in the extracellular matrix composition, such as accumulation of collagen type I is critical in CRC progression [4] and the expression levels of COL1A1 and COL1A2 genes are associated with decreased patient survival (Fig S4a). Oncomine database analysis of the TCGA data set indicated that CRC patients show an elevated COL1A1 and COL1A2 level compared to healthy controls (Fig S4b). Patient-derived CRC organoids started to invade ECM when cultured in collagen I with a parallel increase in the expression of the epithelial–mesenchymal transition (EMT) marker vimentin and an enhanced EV secretion at day 2 after plating (Fig. 3a, b). Importantly, at this timepoint, we could not see a massive apoptosis yet (Fig. 3c). Collectively, these data suggest that the ECM composition is critical for EV release in CRC tumorigenesis.

### Apc mutation increases EV release from 3D organoids

**Fig. 4:**
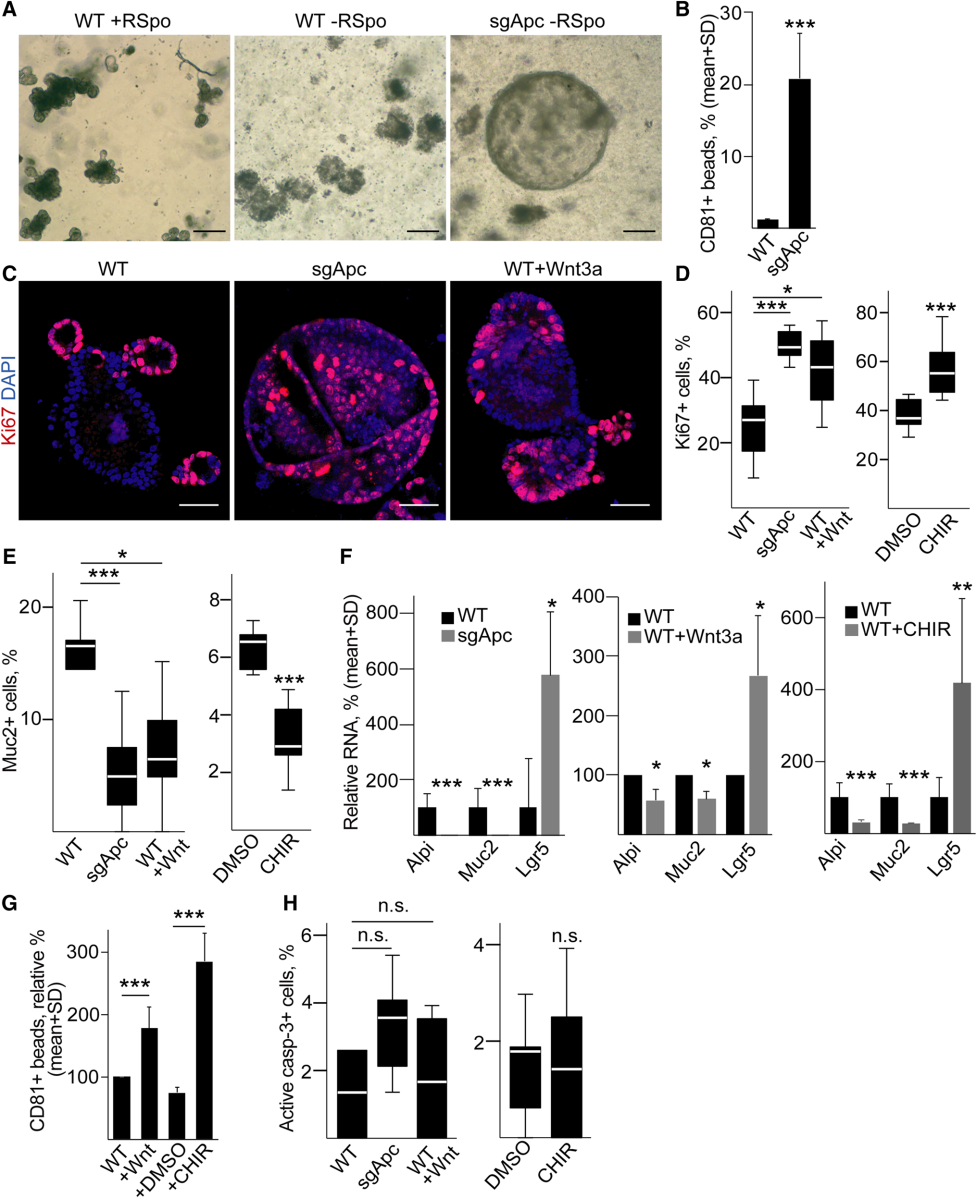
Mouse intestinal organoids release a higher amount of EVs after *Apc* mutation. **a** Morphology of mouse intestinal organoids before (left and middle images) and after *Apc* mutation (right panel). Note that without R-Spondin1 (RSpo) only *Apc*-mutant (right panel), but not wild-type (WT, middle panel) organoids survive. Scale bars: 100 µm. **b** Percentage of CD81 + beads, normalized to equal cell numbers (flow cytometry). Anti-CD81-coated beads were incubated in the supernatant of WT or *Apc*-mutant (sgApc) cultures and EV-bound beads were detected by anti-CD81 (*n* = 4). **c** Confocal microscopic images of WT, *Apc*-mutant and WT organoids treated with Wnt3a (100 ng/ml for 3 days, scale bars: 50 µm). The percentage of Ki67+ (**d**) or Mucin2 + cells (**e**) in the presence of *Apc* mutation or the indicated treatments, counted in confocal microscopic images (*n* = 8–10). In case of organoids treated with CHIR99021 (3 µM for 3 days) that activates the Wnt pathway, results are compared to control samples maintained in 1% DMSO. **f** Relative RNA levels of the indicated genes (qPCR, *n* = 5). **g** Relative percentage of CD81 + beads (flow cytometry). In each case, results were normalized to cell number and they were then compared to the control untreated sample. For all measurements, the Matrigel control samples without organoids gave < 1% positivity for CD81 + beads (*n* = 5). **h** Percentage of active caspase-3 + cells after *Apc* mutation and the indicated treatments, counted on confocal microscopic images (*n* = 10). Student’s paired (F middle panel) or unpaired *t* test (**b** and **f** left and right panels), Kruskal–Wallis test with Dunn post hoc test (**d**, **e**, **h** left panels, **g**) or Mann–Whitney *U* tests (**d**, **e**, **h** right panels) were used

It is widely accepted that EV secretion increases in tumors compared to normal tissues. However, whether this can be attributed to mutations is not yet known. Since CRC cells represent a late stage of tumorigenesis and carry a wide variety of mutations, we used a mouse model with well-defined genetic background. We introduced *Apc* mutation into wild-type (WT) small intestinal organoids by CRISPR-Cas9 and we selected *Apc*-mutant organoids, representing the adenoma stage of intestinal tumors, without adding the external Wnt-agonist R-Spondin1. Since WT intestinal stem cells are strictly dependent on R-Spondin1, only *Apc*-mutant organoids survive after 5 days under these conditions (Fig. 4a). Interestingly, we observed a massive increase in CD81 + EV secretion after *Apc* mutation (Fig. 4b). One possible explanation for the increased EV release after *Apc* mutation is an elevated expression of genes involved in this process. Unfortunately, no single gene is responsible for EV release and different EV pathways may operate in different cell types. Exosomes (EX) are specific EVs of endosomal origin with the best characterized routes of production and secretion among EV types. To test whether the EX route is responsible for the enhanced EV secretion, we selected 39 genes with proved function in multi-vesicular body (MVB) formation and EX release (e.g., specific Rab proteins) (Table S6). This gene set was used in Gene Set Enrichment Analysis (GSEA) on publicly available expression data derived from organoid libraries (GSE74843) [35]. Importantly, these organoid libraries contain only cells of epithelial origin, thus excluding stroma-derived signals. As expected, we observed a positive enrichment of the Wnt-target gene set and KEGG cell cycle genes in the human adenoma samples compared to normal organoids (Fig. S5a). However, the EX biogenesis gene set showed a negative enrichment (Fig. S5a). To rule out the possibility that the EX biogenesis gene set behaves differentially in humans and mice, we repeated our analysis on expression data sets derived from microdissected WT intestinal epithelial cells and *Apc*-mutant adenomas derived from mouse (GSE422). Again, we found no positive enrichment of the EX biogenesis gene set in the adenoma samples (Fig. S5b), suggesting that the increased EV release after *Apc* mutation is not a consequence of the higher expression level of EX biogenesis genes in this system.

*Apc* mutation induces the Wnt pathway. To test whether Wnt pathway activation may lead to an enhanced EV release, WT intestinal organoids were treated with Wnt3a or the GSK-3 inhibitor CHIR99021, known activators of this signaling pathway. Similar to *Apc* mutation, both Wnt3a and CHIR99021 resulted in an increased ratio of Ki67 + proliferating and decreased percentage of Mucin-2 + Goblet cells, which was paralleled by a lower RNA level of Mucin-2 and the enterocyte marker Alpi, and an increase in the Wnt-target stem cell marker Lgr5 (Fig. 4c–f). Importantly, both Wnt3a and CHIR99021 resulted in a massively elevated EV release from organoids, similar to *Apc* mutation (Fig. 4g), without an increased number of apoptotic cells (Fig. 4h). Collectively, these results strongly support the hypothesis that *Apc* mutation increases EV secretion via activating the Wnt pathway in the organoids. Furthermore, since *Apc* mutation is a very early event in intestinal tumorigenesis, our data suggest that increased EV release occurs already in the adenoma stage.

### Fibroblast-derived EVs increase the number of organoids under hypoxia

Since our data show that several factors induce EV release from adenoma and CRC cells, we then applied the organoid system to study the role of EVs in the tumor–stroma communication. First we tested the effect of CRC organoid-derived EVs after differential UC using commercially available normal human colon fibroblasts. We proved the difference in EV amount in the EV-enriched pellet and the EV-depleted supernatant by anti-CD63 or anti-CD81-coated beads and flow cytometry (Fig. S6a). ATCC-1459 human colon fibroblast cells were treated with EV-enriched or EV-depleted medium prepared from supernatants of the 3D CRC organoids and microarray experiments were carried out. In this experimental setting, the effect of EVs was compared to the EV-free CRC-derived condition. As control, organoid-free Matrigel-derived medium (pelleted and supernatant) was used. As expected, CRC-derived medium, independently of the EV content, resulted in an overall change in the gene expression of fibroblasts (Fig. S6b). Interestingly, CRC-derived EVs had no major effect on the transcriptional profile of fibroblasts and a small subset of genes (about 100 genes) showed only a modest (< twofold) expression change (Table S7). To test whether CRC organoid-derived EVs have a critical role under unfavorable conditions, we compared normoxia and hypoxia. CRC organoid-derived EVs did not induce an increased fibroblast motility in wound healing assays in either conditions (Fig. S6c, d). Genes associated with fibroblast activation (ENC1, ST6GALNAC5, SEMA5A, TNFSF4) [36] typically showed a transcriptional increase in EV-depleted CRC organoid medium-treated fibroblasts in hypoxic conditions; however, we detected no additional fibroblast activating effect when EV-enriched medium was added in normoxia or hypoxia (Fig. S6e).

**Fig. 5:**
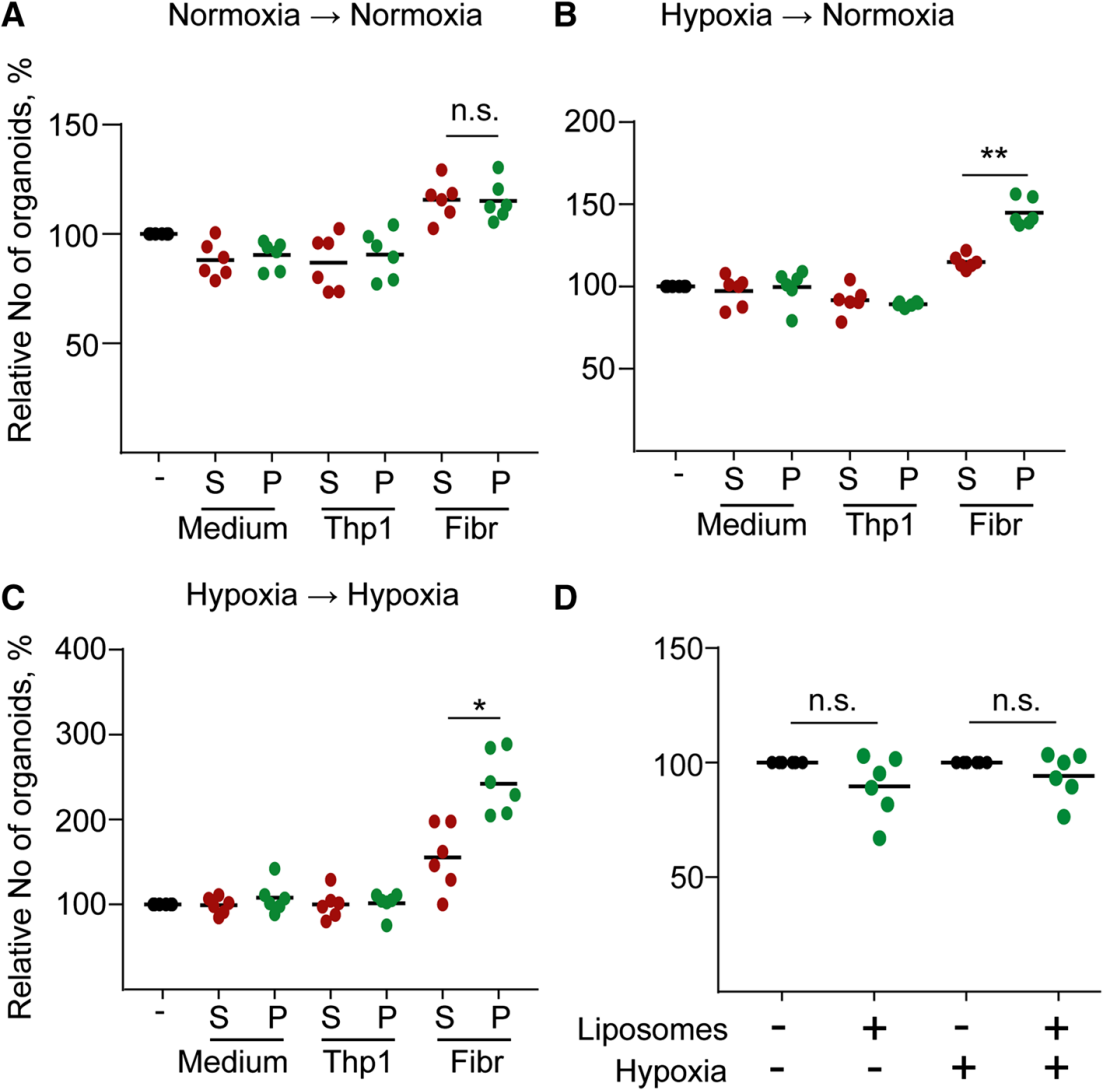
EVs from hypoxic fibroblasts induce the organoid formation of CRC cells. Medium from Thp-1 cells or fibroblasts (Fibr) were collected after 2 days, ultracentrifuged and the supernatant (S) or the pellet (P) were added. Note that in case of cell cultures, the pellet contains the EVs. As control, medium was applied. Samples from normoxic (**a**) or hypoxic conditions (**b**, **c**) were then tested using CRC organoid cells and colony formation was counted as readout either in normoxia (**a**, **b**) or hypoxia (**c**) after 4 days. Three CRC organoid lines were tested twice (***p < 0.005 for all three data sets with Kruskal–Wallis test. Dunn post hoc test was carried out and results from pairwise comparisons are shown only for the indicated comparison). **d** Number of new organoids after treatment with 1 × 10^11^ liposomes. In each experiment, results were compared to the untreated control. Note that liposomes were incubated in normoxia or hypoxia for 2 days, added to CRC organoid cells and the organoid formation was tested in normoxia or hypoxia, respectively

Next, we purified EVs from human colon fibroblasts grown under normoxia or hypoxia and added them to CRC organoid-derived cells in either conditions. Interestingly, we observed an increased number of new organoids after EV treatment only when fibroblasts were cultured in hypoxia (Fig. 5a, c), which was not detected with EV-free supernatant. Importantly, EVs isolated from the acute monocytic leukemia patient-derived THP-1 cell line or liposomes had no effect (Fig. 5a, d), suggesting that the increase in organoid forming efficiency is connected to fibroblast EVs. Collectively, the organoid model system suggests that critical CRC progression factors enhance EV secretion from tumor cells. However, in the tumor–stroma communication, the stromal fibroblast-derived EVs exert a more important role in tumorigenesis.

## Discussion

We show here that smaller EVs are detectable in the supernatant of 3D spheroids, independently of the culture matrix. We provide evidence that patient-derived organoids produce EVs that can be analyzed in the culture supernatant, thus providing evidence that Matrigel-based 3D organoid cultures are suitable for EV studies in CRC. Our data also demonstrate that critical factors in CRC tumorigenesis, such as *Apc* mutation or collagen I deposition, modify EV release from tumor organoids. Interestingly, whereas fibroblast-derived EVs induced the colony formation ability of tumor cells in hypoxia, we could not detect a major effect of organoid EVs on the activation of fibroblasts.

So far, most studies used 2D cell cultures to unravel the functions of EVs in CRC and other cancers. However, the use of different cell lines and different culture conditions resulted in data with low consistency. This may be explained by the fact that these cell lines represent different patients, and during 2D culture establishing protocols, there was a selective advantage for the rapidly proliferating tumor cells that adapted to 2D conditions well. The recently developed 3D organoid technology represents a superior model that maintains the cellular heterogeneity of in vivo tumors without mesenchymal cells [13, 20, 31, 35]. In addition, their culture conditions are standardized and optimized for the best representation of this heterogeneity. To our knowledge, this is the first report of applying the 3D organoid technology for EV analysis in CRC using direct patient-derived samples. In a previous study, Tauro BJ et al. used liquid 3D cell line-derived cultures with no significant loss in cell survival and they identified two distinct exosome populations [37]. Eguchi T et al. used a matrix-free 3D model system for cell lines [38]. Interestingly, when comparing 3D cultures, we detected an increased proportion of apoptotic cells in suspension compared to cultivation in Matrigel, although the size of the apoptotic population was cell line-specific. Thus, 3D suspension culture may be an alternative only for some, but not all of the cancer cell lines and patient-derived organoids. Interestingly, whereas in cell line-derived 3D cultures CD63 + EVs dominate, organoid cultures produce more CD81 + vesicles, thus raising the possibility that EVs released in cell line and organoid-based systems can be different. This is in line with the previous studies showing that EV markers differ between cell types and no universal molecule for EV detection is available [39]. However, whether these EVs functionally differ from each other, still needs to be addressed.

Different cell lines release EV subpopulations at varying levels and the EV subpopulations may have divergent functions due to their different biogenesis and molecular cargo [40–42]. Although the molecular composition of EVs is influenced by the posttranslational modifications of proteins [43], other mechanisms are much less well known. Since there are no genetic models to block the release of only one specific EV subpopulation, recently, EVs have been suggested to be classified based on size (e.g., small and large EV categories) [6]. Importantly, in contrast to 2D and 3D suspension cultures, supernatants of colonies and organoids in 3D matrix contain preferentially smaller EVs.

Although in vivo and in vitro studies show an elevated EV release from tumor cells [11, 44, 45], factors responsible for this effect have been addressed only in a few reports. For example, the presence of p53 is critical for the increased EV release after DNA damage in lung cancer cell lines [46]. *KRAS* mutations represent another important genetic event in many cancer types; in CRC, studies on mutant *KRAS* mostly focused on changes in the molecular cargo of EVs and showed the selective packaging of some RNAs [47, 48]. Thus, our findings that EV release is induced from organoids after *Apc* mutation, a critical genetic event in CRC, and in the presence of collagen I, an ECM protein that often accumulates in CRC, are of particular interest when EV-based diagnostic tools are developed to monitor tumor-derived EVs from body fluids. Furthermore, our studies suggest that *Apc* mutation exerts its enhancing effect on EV release via inducing the Wnt pathway. Wnt activity and cell proliferation are closely connected in the intestinal epithelium and intestinal adenomas. However, since all our data are normalized to cell number, the increased cell number cannot explain the higher EV release. Of note, further studies are required to determine whether the higher EV secretion is a direct effect of the Wnt pathway or it reflects the Wnt-induced changes in the relative ratios of different cell populations.

Stromal fibroblasts critically contribute to CRC tumorigenesis by producing hepatocyte growth factor (HGF) to establish the cancer stem cell phenotype [49] and TGFβ treatment activates fibroblasts to enhance metastasis, partially via IL-11 [5]. Furthermore, there is a strong correlation between the stromal gene expression pattern and the disease outcome in CRC [50]. CRC cell-derived EVs polarize tumor-associated macrophages by miR-145 [51], thus showing that these EVs may be active in some cell populations. Interestingly, in contrast to breast cancer, where cancer cell-derived EVs mediate the stromal mobilization of Wnt–planar cell polarity signaling leading to tumor cell migration [52], we could not detect a marked effect of organoid-derived EVs on fibroblast activation in CRC. However, fibroblast EVs had an enhancing effect on CRC organoid colony-forming ability in hypoxia. The importance of EVs of fibroblast origin is further supported by Hu et al. [53], who found that fibroblast-derived EVs contribute to chemoresistance in CRC. In another study, cancer-associated fibroblast EVs supported pancreatic cancer aggressiveness only in hypoxia and lipid starvation [54], thus showing that EVs may have a function only under unfavorable conditions in some cancer models.

In conclusion, using the novel 3D organoid model, we present evidence that critical factors in CRC initiation and progression, such as *Apc* mutation, induce EV release from tumor cells. Furthermore, fibroblast-derived EVs enhance the colony-forming ability of CRC organoid cells in hypoxia. Our results highlight the power of the organoid technology in EV characterization studies and they provide clues for using EVs as diagnostic markers in CRC.

## Electronic supplementary material

Below is the link to the electronic supplementary material.

Supplementary material 1 (PDF 4958 kb)

Supplementary material 2 (XLSX 22 kb)
