# Supplementary material for: Extracellular vesicle release from intestinal organoids is modulated by Apc mutation and other colorectal cancer progression factors
Source: Cell Mol Life Sci. 2019 Apr 26;76(12):2463–76. doi: 10.1007/s00018-019-03052-1 (PMC6529386; doi:10.1007/s00018-019-03052-1)
Supplement: Supplementary file 1 — Supplementary material 1 (PDF 4958 kb) [file 18_2019_3052_MOESM1_ESM.pdf]

Extracellular vesicle release from intestinal organoids is modulated by Apc mutation and other colorectal cancer progression factors

Supplementary information

Zsuzsanna Szvicsek, Ádám Oszvald, Lili Szabó, Gyöngyvér Orsolya Sándor, Andrea Kelemen, András Áron Soós, Krisztina Pálóczi, László Harsányi, Tamás Tölgyes, Kristóf Dede, Attila Bursics, Edit I Buzás, Anikó Zeöld, Zoltán Wiener

**miR**	**Assay_ID**
mir-10a-5p	479241_mir
miR-16-5p	477860_mir
miR-21-5p	477975_mir
miR-26a-5p	477995_mir
miR-34a-5p	478048_mir
mir-128-3p	477892_mir
mir-146a-5p	478399_mir
miR-155-5p	483064_mir
mir-181a-5p	477857_mir
mir-181b-5p	478583_mir
mir-210-3p	477970_mir
mir-222-3p	477982_mir
mir-532-5p	478151_mir
mir-484	478308_mir

**Primer name**	**Sequence**
mMuc2-fw	TGCTATGTGCCTGGCTCTAA
mMuc2-rev	GCAGGTGGGTTCTTCTTCAG
mAlpi-fw	GGAACAAGAAGGCAGCTGAA
mAlpi-rev	GGGTGTCTCCGGTCCTAGAT
mLgr5-fw	CCTGTCCAGGCTTTCAGAAG
mLgr5-rev	CTGTGGAGTCCATCAAAGCA
mHprt1-fw	GCGATGATGAACCAGGTTATGA
mHprt1-rev	GCCTCCCATCTCCTTCATGA

Table S1. Advanced TaqMan miRNA assays and the sequence of primers used to quantify mRNA levels by RT-qPCR.

Sample	Sex	Age	Tumor	Differentiation grading	Stage	MMR deficiency
CRC1	F	63	colon adenocarcinoma	Grade 2	T3N0M0	no
CRC2	M	74	colon adenocarcinoma	Grade 2	T3N0M0	no
CRC3	M	75	colon adenocarcinoma	Grade 2	T3N2aM0	no
CRC4	M	74	colon adenocarcinoma	Grade 2	T3N1M0	no
CRC5	F	70	colon adenocarcinoma	Grade 2	T3N0M0	no

Table S2. Clinical data of CRC patients. MMR deficiency was determined by immunostaining of the samples for MLH1, MSH2, PMS2 and MSH6 in clinical diagnosis.

Sample	p53 mutation	Nutlin sensitivity	KRAS mutation*	EGF dependency
CRC1	V274A	no	no	yes
CRC2	V274A	no	no	yes
CRC3	V274A	no	no	yes

Table S3. Characteristics of the CRC patient-derived organoids.*Only codon 12, 13, 18 and 61 were tested for KRAS. Note that none of the cultures survived in the absence of R-Spondin1.

Symbol	Description
CFAI	Complement factor I OS=Homo sapiens GN=CFI PE=1 SV=2
DHE3	Glutamate dehydrogenase 1, mitochondrial OS=Homo sapiens GN=GLUD1 PE=1 SV=2
EPCAM	Epithelial cell adhesion molecule OS=Homo sapiens GN=EPCAM PE=1 SV=2
FPRP	Prostaglandin F2 receptor negative regulator OS=Homo sapiens GN=PTGFRN PE=1 SV=2
HEMO	Hemopexin OS=Homo sapiens GN=HPX PE=1 SV=2
HSP7C	Heat shock cognate 71 kDa protein OS=Homo sapiens GN=HSPA8 PE=1 SV=1
IGHA1	Immunoglobulin heavy constant alpha 1 OS=Homo sapiens GN=IGHA1 PE=1 SV=2
IGHG1	Immunoglobulin heavy constant gamma 1 OS=Homo sapiens GN=IGHG1 PE=1 SV=1
IGHG4	Immunoglobulin heavy constant gamma 4 OS=Homo sapiens GN=IGHG4 PE=1 SV=1
IGKC	Immunoglobulin kappa constant OS=Homo sapiens GN=IGKC PE=1 SV=2
IGLC3	Immunoglobulin lambda constant 3 OS=Homo sapiens GN=IGLC3 PE=1 SV=1
ITIH4	Inter-alpha-trypsin inhibitor heavy chain H4 OS=Homo sapiens GN=ITIH4 PE=1 SV=4
LAMA1	Laminin subunit alpha-1 OS=Homo sapiens GN=LAMA1 PE=1 SV=2
LAMB1	Laminin subunit beta-1 OS=Homo sapiens GN=LAMB1 PE=1 SV=2

LAMC1	Laminin subunit gamma-1 OS=Homo sapiens GN=LAMC1 PE=1 SV=3
LG3BP	Galectin-3-binding protein OS=Homo sapiens GN=LGALS3BP PE=1 SV=1
NID1	Nidogen-1 OS=Homo sapiens GN=NID1 PE=1 SV=3
OLFM4	Olfactomedin-4 OS=Homo sapiens GN=OLFM4 PE=1 SV=1
PDC6I	Programmed cell death 6-interacting protein OS=Homo sapiens GN=PDCD6IP PE=1 SV=1
PGBM	Basement membrane-specific heparan sulfate proteoglycan core protein OS=Homo sapiens GN=HSPG2 PE=1 SV=4
SDCB1	Syntenin-1 OS=Homo sapiens GN=SDCBP PE=1 SV=1
TRFE	Serotransferrin OS=Homo sapiens GN=TF PE=1 SV=3

Table S4. List of proteins present in all three CRC organoid samples (org 1-3), but absent in the empty Matrigel controls. See also **Figure S3A** and **Table S5** (separate Excel file).

**Gene Symbol**	**Gene Symbol**	**Gene Symbol**
CD63	RAB27B	STAM
CD81	RAB2B	TSG101
CD9	RAB35	VAMP7
CHMP2A	RAB5A	VPS25
CHMP3	RAB7	VPS28
CHMP4A	RAB9	VPS32
CHMP4B	RALA	VPS36
CHMP6	SDCBP	VPS37A
HGS	SDCBP2	VPS37B
PDCD6IP	SMPD1	VPS37C
PLD2	SMPD2	VPS4A
RAB11A	SMPD3	VPS4B
RAB27A	SNF8	VTA1

Table S6. The EX biogenesis gene set. Genes with known function in EX biogenesis / secretion were collected from the literature**.**

**ID**	**Symbol**	**p-value**	**Fold change (EV/noEV)**
A_33_P3263538	NA	0.000309	1.543993487
A_33_P3382105	NA	2.00E-06	1.474269217
A_33_P3345743	NA	2.50E-05	1.391524844
A_23_P164022	MYO1C	1.20E-05	1.378723669
A_33_P3219697	NA	5.30E-05	1.375541818
A_33_P3802146	NA	3.40E-05	1.372367311
A_33_P3265606	NA	2.40E-05	1.353473524
A_33_P3209176	NA	2.20E-05	1.3441244
A_33_P3269803	NA	0.000672	1.337927555
A_23_P52336	UNC5B	0.000596	1.334839854
A_33_P3645805	NA	0.000399	1.325619442
A_23_P109907	ILDR1	0.00011	1.322560146
A_23_P214168	COL12A1	0.000715	1.322560146
A_24_P734953	TRNP1	8.80E-05	1.322560146

A_23_P60079	ANGPT2	0.000106	1.319507911
A_23_P382199	VAPA	0.000105	1.31646272
A_23_P146077	ZNF395	7.80E-05	1.310393404
A_33_P3246883	NA	0.000128	1.304352069
A_33_P3451157	NA	0.000129	1.295342252
A_23_P152858	COPRS	0.000523	1.292352831
A_23_P303718	DST	0.000513	1.289370309
A_33_P3239267	NA	0.00043	1.289370309
A_33_P3421219	NA	0.000215	1.289370309
A_33_P3752697	NA	0.000404	1.289370309
A_23_P52727	NAV2	0.000496	1.286394669
A_23_P69908	GLRX	0.000196	1.283425898
A_33_P3259557	NA	0.000163	1.280463977
A_33_P3294961	NA	0.000179	1.277508892
A_33_P3353051	NA	0.000194	1.277508892
A_23_P79927	NOP56	0.000156	1.271619167
A_24_P331704	KRT80	0.000288	1.271619167
A_33_P3338961	NA	0.000492	1.271619167
A_32_P41375	LINC01000	0.000204	1.268684494
A_33_P3280066	NA	0.000493	1.268684494
A_23_P59602	MIOS	0.000291	1.265756594
A_23_P73780	IRAK1	0.000363	1.265756594
A_24_P370887	VAMP3	0.000494	1.265756594
A_33_P3321781	NA	0.000175	1.265756594
A_33_P3364696	NA	0.000273	1.262835451
A_23_P135616	STX18	0.000411	1.25992105
A_23_P28466	DAW1	0.000484	1.25992105
A_23_P69586	FAT1	0.000385	1.25992105
A_23_P75380	AIP	0.000385	1.25992105
A_24_P304051	GSTO1	0.000859	1.25992105
A_33_P3241596	NA	0.00067	1.25992105
A_23_P201342	DVL1	0.000574	1.257013375
A_23_P162945	SRP54	0.000356	1.25411241
A_33_P3234487	NA	0.000875	1.25411241
A_33_P3262191	NA	0.00043	1.25411241
A_33_P3317253	NA	0.000388	1.25411241
A_24_P252130	PPARD	0.000725	1.251218139
A_33_P3243878	NA	0.000412	1.251218139
A_33_P3243907	NA	0.000388	1.251218139
A_33_P3333033	NA	0.00033	1.251218139
A_24_P116242	KLHDC2	0.000541	1.248330549
A_24_P160413	NA	0.000579	1.248330549
A_33_P3280044	NA	0.000528	1.248330549
A_33_P3422289	NA	0.000373	1.245449623
A_23_P166663	APPL1	0.000771	1.242575344
A_33_P3233841	NA	0.000522	1.242575344
A_33_P3220437	NA	0.000421	1.2397077
A_33_P3292179	NA	0.000541	1.2397077
A_33_P3407324	NA	0.00056	1.2397077
A_23_P117971	NA	0.000708	1.236846674
A_23_P145863	S100A11	0.000596	1.236846674
A_23_P87742	IFFO1	0.000871	1.236846674
A_33_P3397795	NA	0.000612	1.236846674

A_23_P14083	AMIGO2	0.000546	1.233992249
A_33_P3265956	NA	0.000627	1.233992249
A_33_P3289561	NA	0.000733	1.233992249
A_33_P3354499	NA	0.000805	1.231144413
A_24_P51588	CCNL2	0.000582	1.22830315
A_33_P3362321	NA	0.000948	1.22830315
A_33_P3385765	NA	0.000856	1.225468442
A_23_P161634	MUS81	0.000942	1.222640278
A_23_P395595	FNBP4	0.00095	1.222640278
A_33_P3304382	NA	0.000752	1.222640278
A_33_P3371718	NA	0.000854	1.222640278
A_33_P3384442	NA	0.000854	1.222640278
A_23_P208516	MBOAT7	0.000894	1.21981864
A_33_P3212823	NA	0.000776	1.21981864
A_33_P3759592	NA	0.000853	1.21981864
A_33_P3503937	NA	0.000892	1.217003513
A_33_P3326904	NA	0.000935	1.214194884
A_23_P54991	DYNLL2	0.000995	0.816014485
A_23_P19712	GMNN	0.000919	0.804780173
A_24_P117029	LDLR	0.000581	0.802922882
A_33_P3293524	NA	0.000789	0.79922115
A_23_P132277	MCM5	0.000435	0.795536484
A_33_P3368830	NA	0.000327	0.788218036
A_33_P3420446	NA	0.000643	0.784584098
A_23_P387471	MICB	0.000138	0.775572381
A_23_P20443	LZTS1	5.70E-05	0.759611332
A_33_P3247086	NA	3.80E-05	0.759611332
A_23_P30495	HMGCR	6.10E-05	0.742261785
A_33_P3382887	NA	0.000114	0.723634619

Table S7. Differentially regulated genes between fibroblasts treated with EV-enriched and EV-depleted, CRC-derived medium. Cells were treated for 2 days ($p<0.001$, no multiple testing correction).

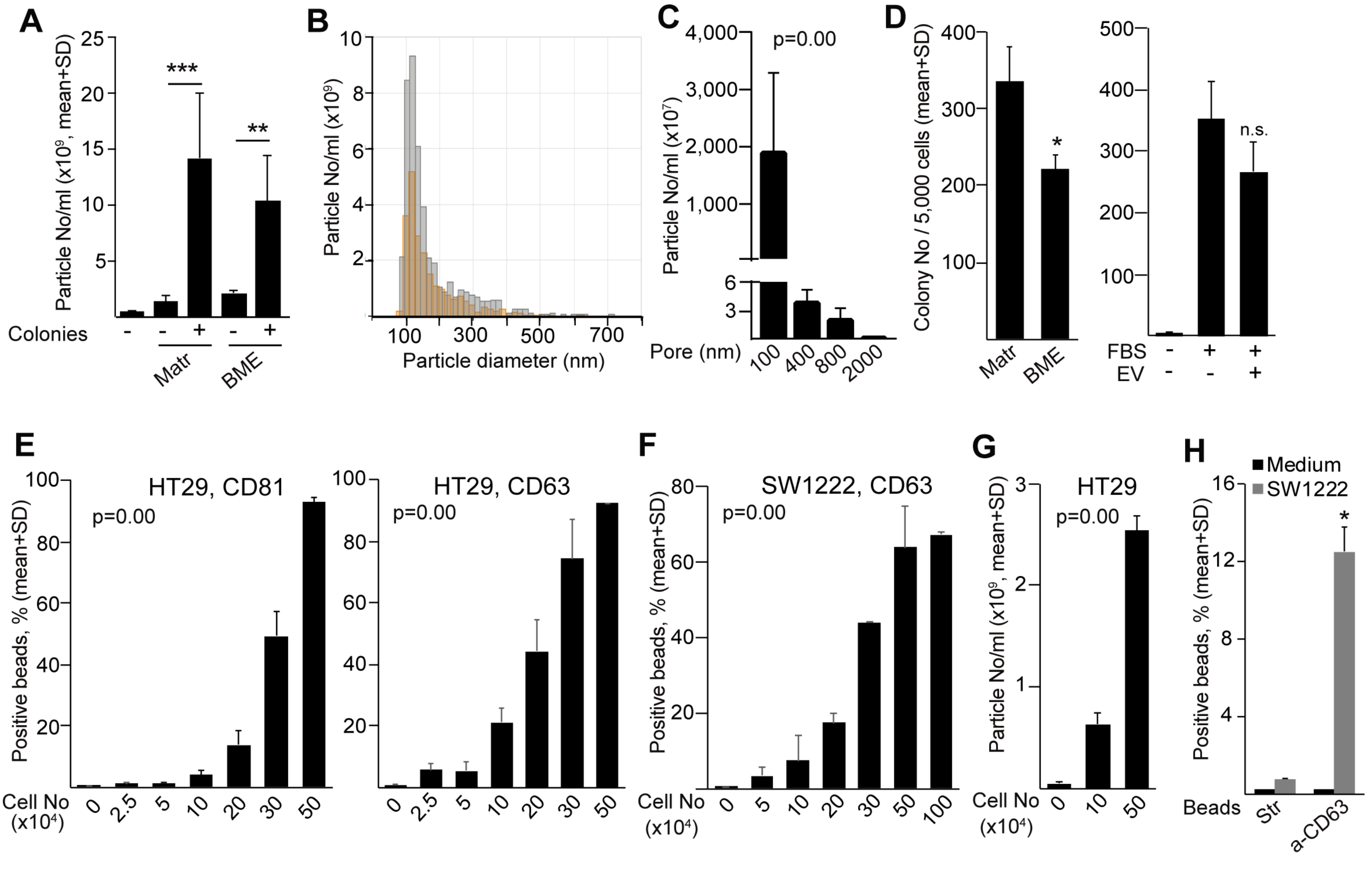

Figure S1. EVs derived from colonies can be detected in cultures in 3D matrix. A) Particle number of the medium, supernatant derived from Matrigel or BME matrix, with or without equal numbers of SW1222 cells. Colonies were cultured for 12 days and after changing the medium, samples were collected at 48 h (qNano, 100 nm pore size, n=3, paired t-test). B) The distribution of particle size in supernatant derived from SW1222 colonies after 12 days in Matrigel (grey) or BME (orange). Samples for measurements were collected after 48 h. C) Particle concentrations measured with NanoPore membranes of different pore sizes (qNano, n=4, ANOVA). Supernatants from SW1222 colonies grown in Matrigel were collected after 48 h. The background particle number in Matrigel control was substracted from the values for each pore size. (D) Colony forming efficiency of SW1222 cells in Matrigel or BME (left panel) or in Matrigel with or without 2.5% EV-free FBS (n=3, paired t-test). E-F) The percentage of positive beads in 2D HT29 (E) or SW1222 (F) cultures with increasing cell numbers (flow cytometry, n=2-3, ANOVA). Since in case of SW1222 cultures, the percentage of CD81+ beads was below 5% even at a high cell number (1 x 106 cells), only results with anti-CD63-coated beads were used. G) Particle concentration at different cell numbers in HT29 cell cultures (qNano, 400 nm pore size, n=3, ANOVA). H) The percentage of positive beads coated with anti-CD63 or Streptavidin (Str) and detected by anti-CD63. Beads were incubated in 3D culture supernatant with (SW1222) or without (medium) colonies (flow cytometry, n=3, t-test). In all experiments, 400 µl supernatant from the indicated cell number was used for EV detection.

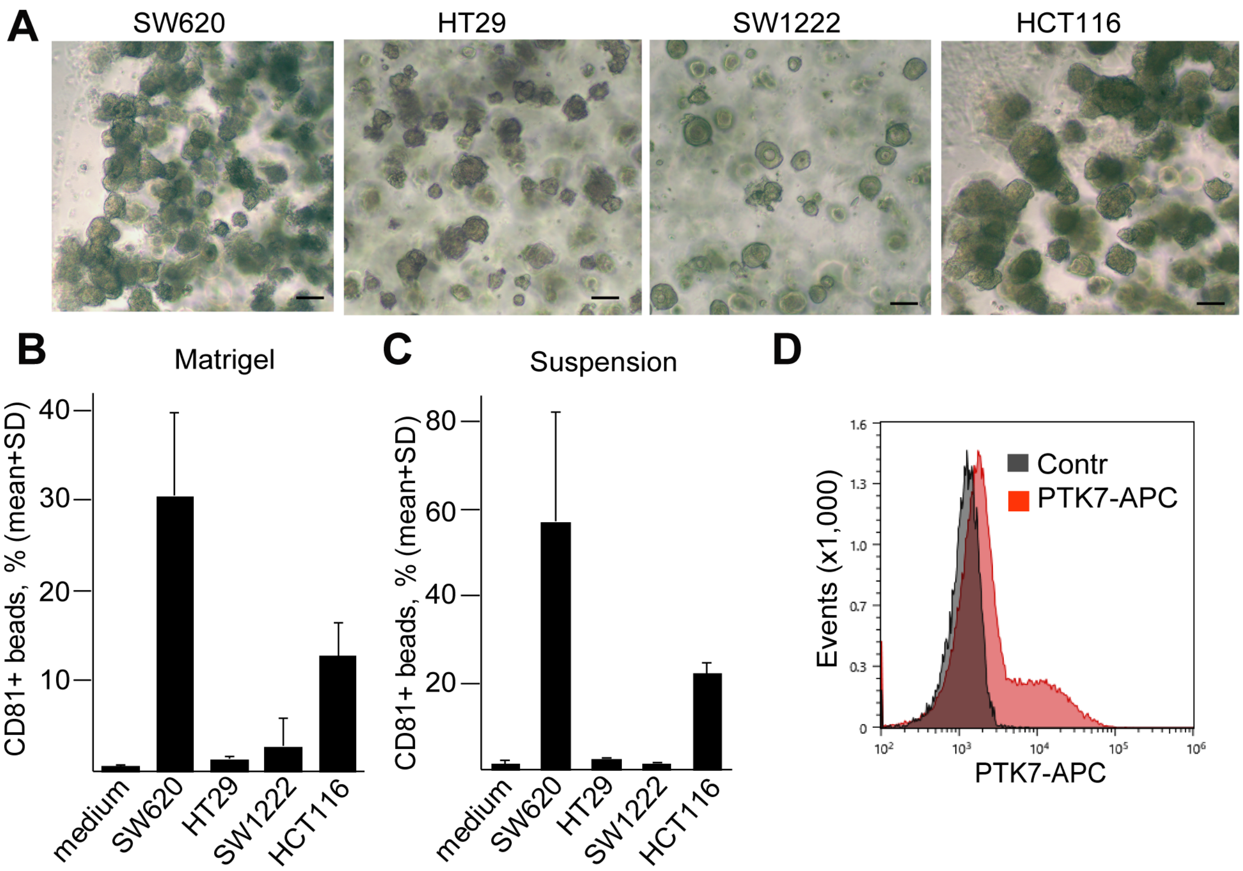

Figure S2. CD81+ EVs can be detected in the medium of 3D CRC colony cultures. A) The morphology of 3D colonies derived from the indicated cell lines (scale bar: 50 µm). B-C) Percentage of CD81+ beads incubated in the supernatant of Matrigel cultures (B) or suspension cultures (C). Data are normalized to 500,000 cells (flow cytometry, n=3). D) Representative flow cytometric measurement of cell surface PTK7 expression in CRC organoid-derived cells (control: isotype control sample).

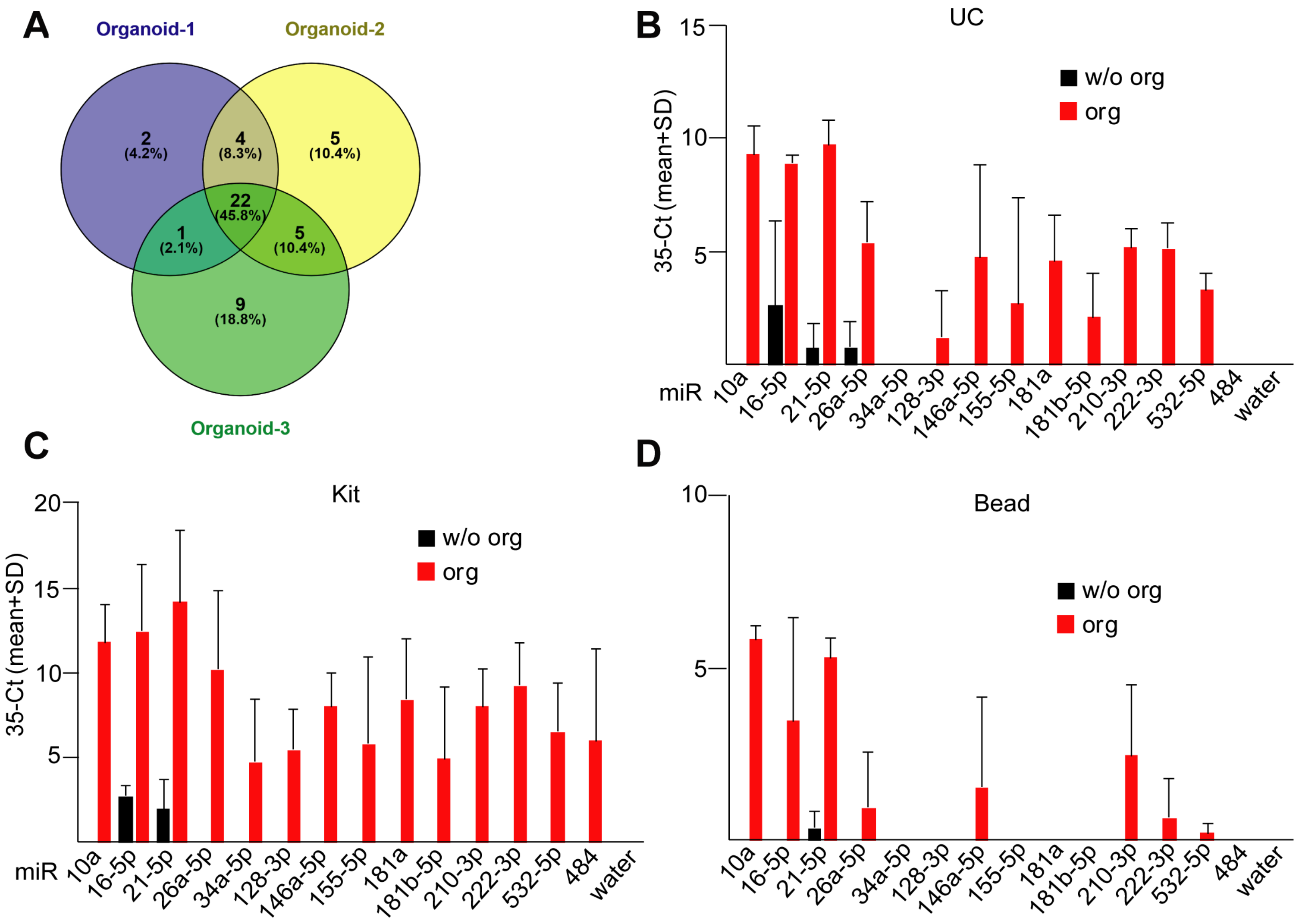

Figure S3. Proteomic characterization and miRNA profiling of CRC organoid-derived EVs. A) Venn diagram of the detected proteins in three CRC organoid lines. See also Table S4 and S5. B-D) 35-Ct values for the indicated miRNAs after isolating EVs with different methods. UC: serial centrifugation and ultracentrifugation (B), Kit: ExoRNEasy Serum/Plasma Starter Kit (Qiagen (C)), Bead: anti-CD63-coated beads (D). In each case, equal cell numbers and culture volumes were used in the experiments (TaqMan assay, n=3).

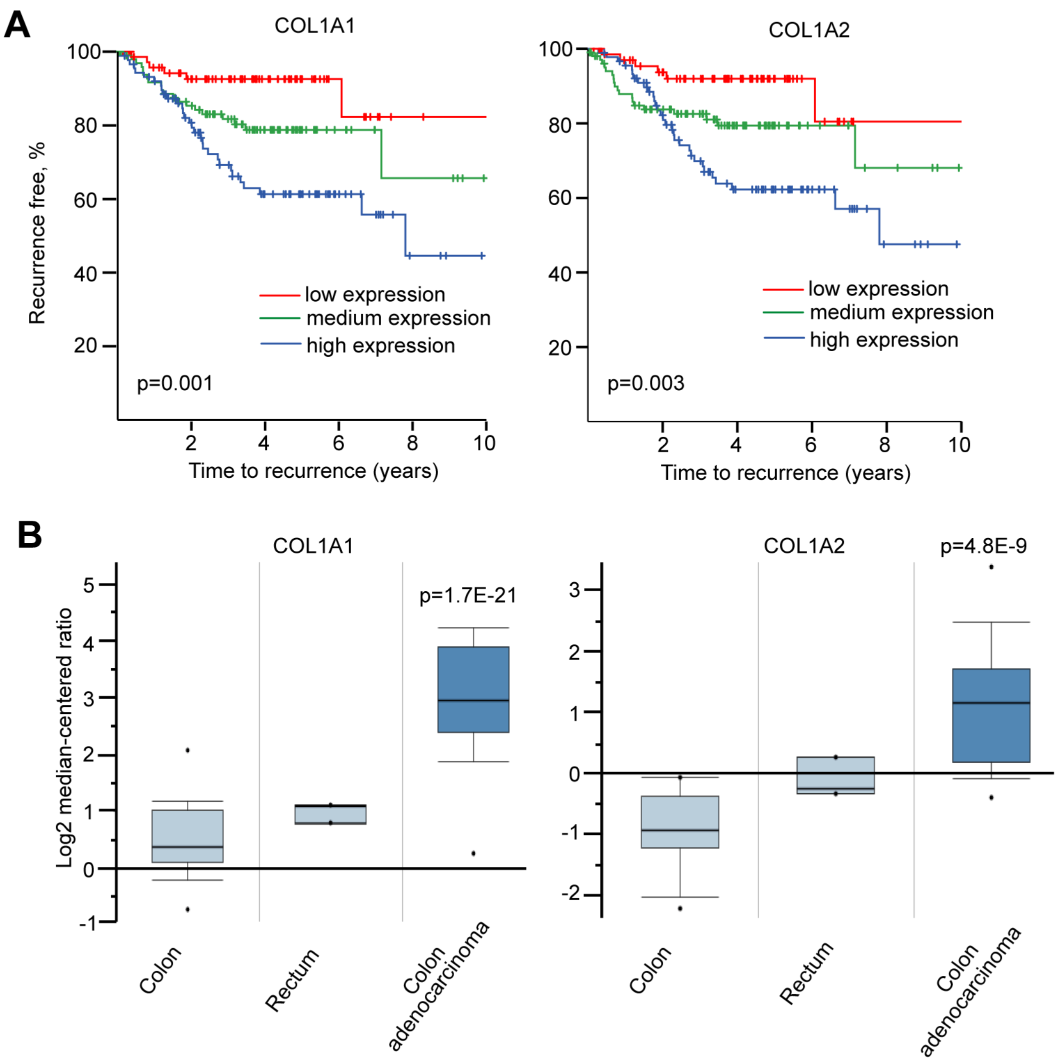

Figure S4. Survival and expression level analysis of the COL1A1 and COL1A2 genes encoding collagen I. A) Kaplan-Meier survival plot for the indicated genes (low expression: z-score normalized data <-0.5*SD, high expression group: expression level > 0.5*SD, medium: -0.5*SD < z-score normalized value < 0.5*SD). p-value derived from log rank test is shown (probe ID for COL1A1: 202311_s_at, for COL1A2: 202404_s_at). B) Normalized expression level of the indicated genes in the TCGA gene set (Oncomine analysis, www.oncomine.org, reporter A_23_P207521 for COL1A1 and A_23_P255247 for COL1A2 are shown. Note that results were significant for all the reporters).

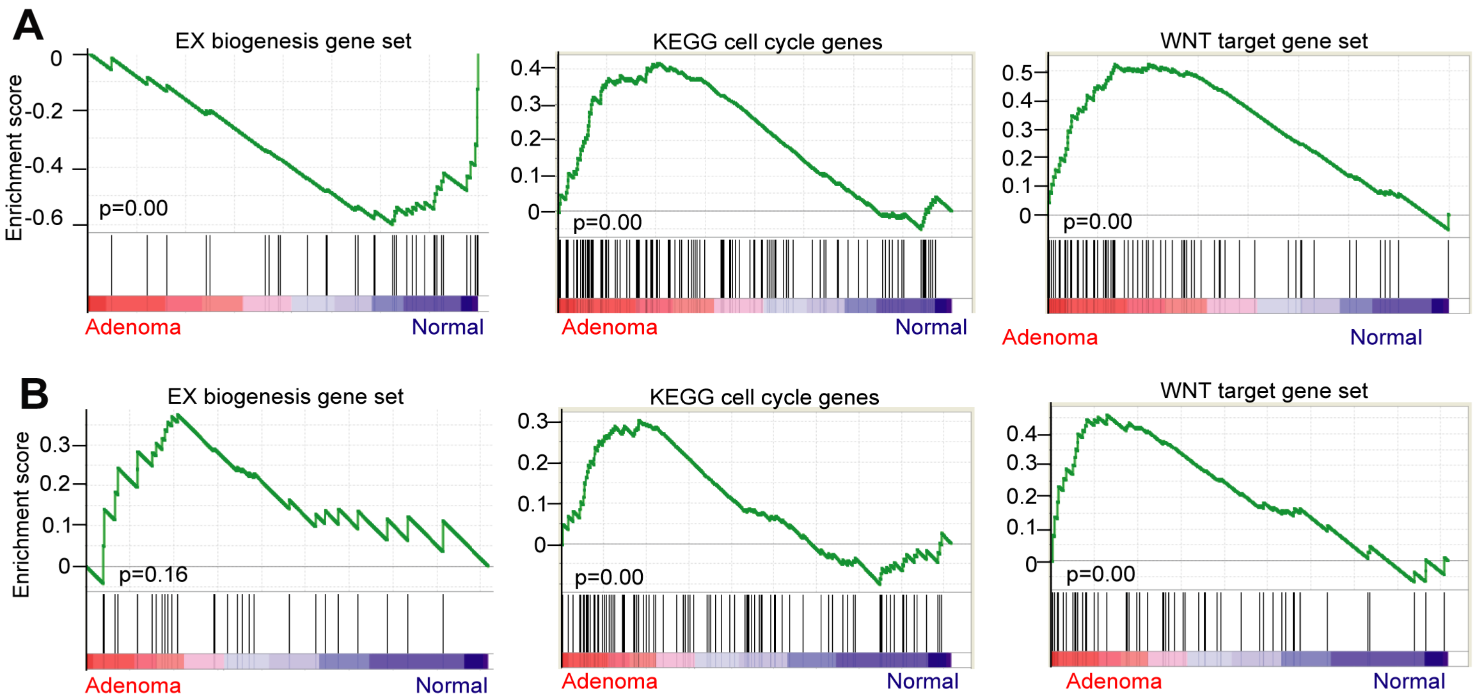

Figure S5. The EX biogenesis gene set shows no positive enrichment in intestinal tumor cells. Gene Set Enrichment Analysis (GSEA) of an expression dataset from human CRC organoid library, containing adenoma samples as well (A) and from mouse intestinal adenoma cells (B). In both cases the human or mouse adenoma samples were compared to WT controls. Note that KEGG cell cycle and Wnt target gene sets showed a positive enrichment (positive controls). Nominal p-values are indicated.

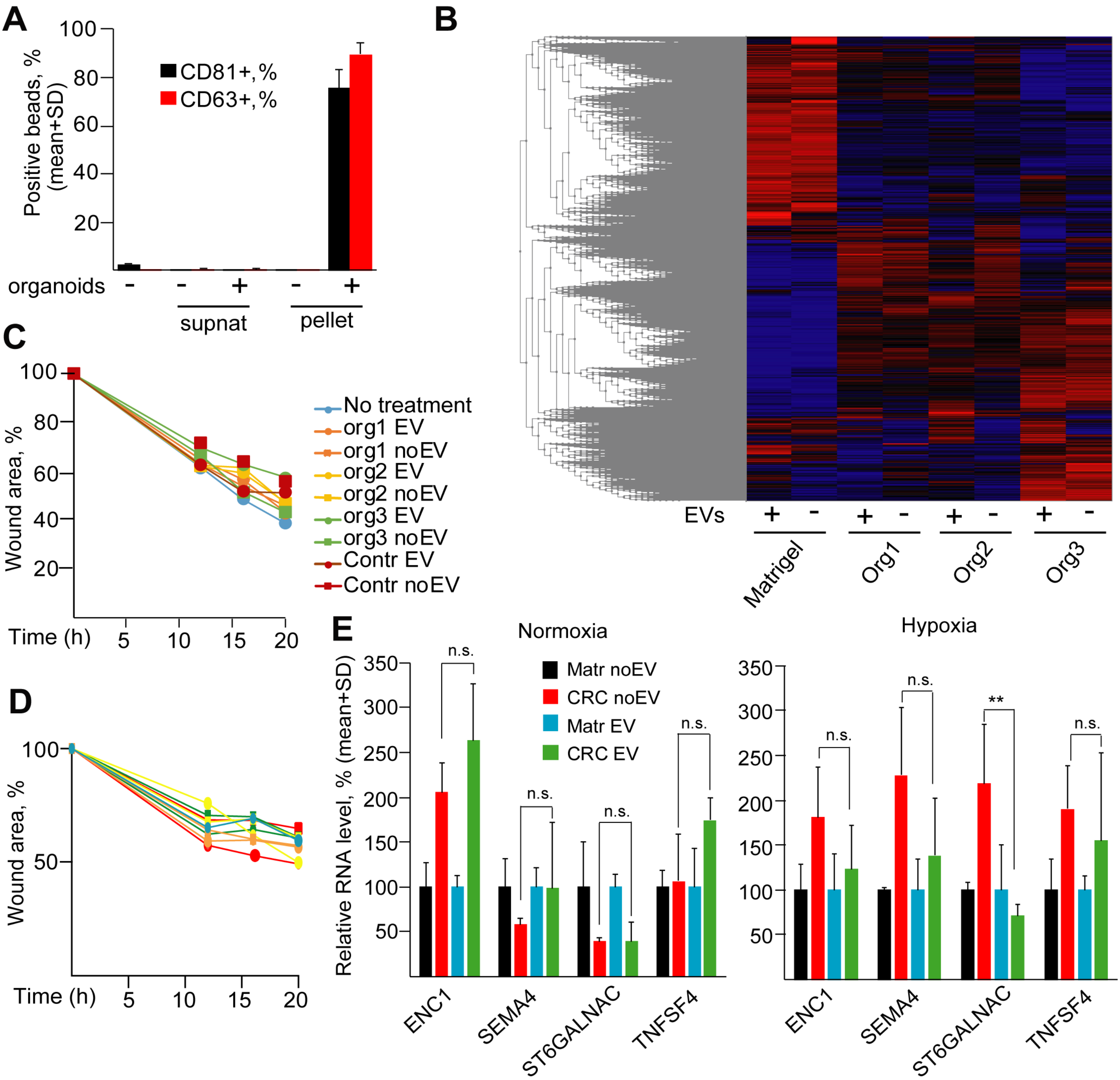

Figure S6. CRC organoid-derived EVs do not activate fibroblasts. A) Percentage of CD81+ and CD63+ beads, detected by anti-CD81 and anti-CD63 antibodies, respectively (flow cytometry). Beads were incubated in the supernatant or resuspended (in PBS) pellet derived from 3D cultures with or without organoids after UC. Note that positive beads were detected only in the pellet of organoid cultures (n=2). B) Hierarchical clustering of microarray data of human colon fibroblasts. Fibroblasts were treated with EV-enriched and EV-depleted medium from Matrigel without or with the indicated organoids (EVs were collected from 300,000 cells and ultracentrifuged). RNA was isolated after 48 h. C-D) Relative wound area compared to the initial area in confluent fibroblast cultures treated with EV-enriched (EV) or EV-depleted (noEV) medium produced with UC from the indicated organoid cultures. Organoids were cultured in normoxia (C) or hypoxia (D). Control: samples without organoids, no treatment: fibroblasts without CRC medium. E) Relative RNA level of genes associated with fibroblast activation. CRC organoid-derived supernatants (cultured in normoxia or hypoxia for 3 days) were collected, ultracentrifuged and fibroblasts were treated with EV-enriched and EV-depleted medium in normoxia or hypoxia for 2 days. Matr: fibroblasts treated with samples without organoids. Note that CRC-derived EVs have no inducing effect (compare red and green bars). RT-qPCR, n=3. ANOVA and Tukey post hoc test, only the significance of the comparison of CRC noEV and CRC EV is shown.
